# Predictive risk factors of steroid dependent nephrotic syndrome in children

**DOI:** 10.15171/jnp.2017.31

**Published:** 2017-02-02

**Authors:** Maher Ahmed Abdel-Hafez, Nagy Mohamed Abou-El-Hana, Adel Ali Erfan, Mohamed El-Gamasy, Hend Abdel-Nabi

**Affiliations:** Pediatric Department, Faculty of Medicine, Tanta University, Egypt

**Keywords:** Nephrotic syndrome, Children, Prediction, Steroid dependency

## Abstract

**Background:**

Development of steroid dependency is one of the difficult problems in the management of children with idiopathic nephrotic syndrome, leading to increased morbidity, complications and cost of treatment. Thus, predicting early in the disease course will be useful in counseling parents and may improve treatment strategy.

**Objectives:**

To determine the clinical characteristics that can predict the development of steroid dependency early in the initial episodes of steroid sensitive nephrotic syndrome (SSNS).

**Patients and Methods:**

The study included 52 children with SSNS. Their ages ranged from 3 to 16 years. Patients were divided into two groups. Group A consisted of 24 patients with steroid dependency or frequent relapses nephrotic syndrome and group B consisted of 28 patients with complete remission or recurrent nephrotic syndrome. Data obtained retrospectively from patients’ files.

**Results:**

Children who require a cumulative steroid dose equal or more than 140 mg/kg to maintain remission during the first 6 months of the disease are at high risk to require steroid sparing agents (SSA) for disease control, and who did not achieve remission by day 20 of the initial prednisone course became steroid dependent with 96% specificity but with low sensitivity (50%). All steroid dependent children in this study showed relapses associated significantly with upper respiratory tract infections.

**Conclusions:**

Cumulative steroid dose in the first 6 months of treatment and the need of more than 20 days to achieve initial remission can predict steroid dependency in children with nephrotic syndrome.

**Implication for health policy/practice/research/medical education:**

The results of this article can help health managers to improve protocols of managements of idiopathic nephrotic syndrome in children by prediction of steroid dependency early in the disease course and can help in parents counsellings as well.

## 1. Background

The prognosis of children with idiopathic nephrotic syndrome depends on the underlying histopathology and can be predicted by the response to steroid treatment. Biopsy has proven focal segmental glomerulosclerosis (FSGS) and steroid resistant nephrotic syndrome (SRNS) are significantly associated with poor outcome including progression to end-stage renal disease (ESRD) (1). Most patients with minimal change disease (MCD) achieve remissions by two weeks of steroid therapy. The disease recurs in more than 75% of subjects and almost half show frequent relapses or steroid dependency (2). The associations of steroid dependency with young age at onset, male gender, the duration from initial steroid therapy to remission, low serum protein levels and hematuria have been investigated with conflicting results in different population groups (3-7). Although the higher incidence of nephrotic syndrome previously has been reported in Arab children (8), little is known about their response to steroid therapy. Predicting high dose steroid dependency in children with nephrotic syndrome will help us to select patients who will benefit from adding steroid sparing agents (SSA) early and avoid possible side effects from prolonged steroid treatment.

## 2. Objectives

To determine the clinical characteristics that can predict the development of steroid dependency early in the initial episodes of steroid sensitive nephrotic syndrome (SSNS).

## 3. Patients and Methods

All children with steroid sensitive idiopathic nephrotic syndrome with regular follow up at outpatient clinic of the pediatric nephrology unit, Tanta University Hospitals were considered for inclusion in this retrospective investigation.

### 3.1. Exclusion criteria

- Children with follow up period less 1 year.
- Children with steroid resistant nephrotic syndrome.
- Children with secondary nephrotic syndrome.

An initial remission followed by two or more relapses during the steroid diminution period or within 15 days after tapering of prednisone was defined as steroid dependency. A relapse was defined by recurrence of severe proteinuria (>40 mg/m^2^/h, or urine albumin dipstick ≥3+ on 3 successive days), often with a recurrence of edema. A complete remission was defined by marked reduction in proteinuria (to <4 mg/m^2^/h, or urine albumin dipstick of 0 to trace for 3 consecutive days) in association with resolution of edema.

Frequent relapsing nephrotic syndrome was defined as two or more relapses in 6 months or three or more relapses in 1 year. Steroid resistance was considered after 6 weeks of daily full dose steroid without response (without improvement of proteinuria).

The study included 52 children with steroid sensitive idiopathic nephrotic syndrome.

Patients were divided into two groups: group A consisted of 24 patients who were steroid dependents or frequent relapses; Group B consisted of 28 patients with complete remission or recurrent nephrotic syndrome but not frequent relapsing.

Data were obtained retrospectively from patient’s history about symptoms of nephrotic syndrome, duration of the disease, response to steroid therapy in the first presentation, frequency of relapses, other medications and complications of therapy. Data obtained from history was confirmed by revising patients’ medical records and the following parameters were analyzed: Age at onset of nephrotic syndrome, gender, presence of atopic disease (asthma, allergic rhinitis, allergic conjunctivitis and/or urticaria either in the patient or in one of his family). Additionally, days to remission after initial steroid therapy, concurrent upper respiratory tract (URT) infections during relapses and proportion of relapses during the period of follow up were recorded. Likewise, cumulative steroid dose received in the first 6 months after the onset of the disease (which was calculated by adding daily or alternate day doses of steroid received during the first 180 days and expressed per kg body weight), immunosuppressive drugs and serum creatinine at onset and at time of the study were also mentioned.

### 3.2. Protocol of treatment

All patients initially at first episode nephrotic syndrome were treated by prednisone (60 mg/m^2^/d) for a full duration of 30 days, while relapses were treated by oral prednisone at 60 mg/m^2^/d until remission plus 5 additional days. Then the prednisone dose was tapered to 40 mg/m^2^ every other day for 4 weeks, followed by a reduction of 10 mg/m^2^ every other day per week to the lowest dose necessary to maintain remission and, ultimately, discontinuation of treatment if appropriate.

Steroid-sparing agents were introduced if the patient presented frequent relapses and/or if the steroid dosage to maintain remission was ≥20 mg/m^2^EOD and/or if steroid related side effects were unacceptable in terms of growth retardation or obesity despite adequate diet. Cyclophosphamide in a dose of 2 mg/kg/d for 3 months was administered to treat frequent relapsing nephrotic syndrome and to decrease the recurrence rate. Cyclosporine 6 mg/kg/d was administered to treat high dose steroid dependent nephrotic syndrome and the dose was adjusted to maintain a target serum level of cyclosporine between 100-150 ng/mL in the first 3 months of therapy and 50-100 ng/ml later on as long as the patients were in remission.

### 3.3. Ethical issues

1) The research followed the tenets of the Declaration of Helsinki; 2) informed consent was obtained, and 3) the research was approved by the ethical committee of Faculty of medicine, Tanta University, Egypt .

### 3.4. Statistical analysis

Statistical analysis of the present study was performed by using SPSS version 17. Data were expressed as the number and percentage for qualitative variable, mean + SD for quantitative variable. Unpaired student *t* test and chi-square were applied to compare between the two groups in quantitative and qualitative data respectively. Receiver operating curve (ROC) characteristic was used to predict steroid dependency.

## 4. Results

Patient characteristics were summarized in Table 1. Patients’ age at time of the study ranged from 3 years to 16 years. There was no significant difference between the two groups as regard age at disease onset or at the time of the study. The study included 52 children (31 males and 21 were females) with SSNS. Their ages ranged from 3 to 16 years. There was no significant difference between both groups in sex distribution. Days from onset of steroid administration to clinical remission were significantly higher in group A, compared to group B (*P* < 0.001). Clinical data at initial presentations including 24 hours urinary proteins, serum albumin, total serum cholesterol, arterial blood pressure and presence of microscopic hematuria were not significantly different between groups.

**Table 1:** Patients’ demographics and clinical characteristics at initial presentation

	**Group A** **(n = 24)**	**Group B** **(n = 28)**	*t*	*P*
Age at disease onset (year)	4.81± 2.1	4.19± 2.03	0.77	0.47
Age at time of the study (year)	8.33±4.08	7.03± 3.06	0.87	0.38
Males/females (%)	15/9	16/12	0.15^a^	0.98
Days to remission^b^	19.96± 7.1	13.2±5.2	3.9	0.001
Serum albumin (g/dL)	2.1±0.4	2.15±0.36	0.81	0.41
24 Hour urinary proteins (mg/m^2^/h)	80.2±25	79.5±24	0.88	0.37
Total serum cholesterol (mg/dL)	320± 75	325±65	0.91	0.21
Mean arterial blood pressure (mm Hg)	74±12	73±10	0.87	0.26
Microscopic hematuria (cell/HPF)	15 (62.5%)	16 (57.1%)	0.19^a^	0.87
Positive history of atopy, n (%)	4 (16.7)	6 (21.4)	0.189^a^	0.664

Abbreviation‏: HPF, high power field. ^a^Chi-Square test; ^b^ From onset of steroid therapy.

Table 2 demonstrates the follow up data of the studied patients. The mean follow up period and the proportion of relapses were significantly higher in steroid dependency/frequent relapses group (SD/FR group). Cumulative steroid dose required to maintain remission in the first 6 months of the disease were significantly higher in the SD/FR group. Relapses in both groups were commonly associated with concomitant URT infections.

**Table 2:** Follow up data of the studied patients

	Group A (n=24)	Group B ( N=28)	*t*	*P*
Follow up duration (years)	3.91±2.16	2.85±1.25	2.1	0.04
Number of relapses during the period of follow up	3.70± 2.09	2.60±1.13	2.403	0.020
Cumulative steroid dose during the first 6 months of the disease (mg/kg)	160 ± 21	95± 25	7.8	<0.001
Concomitant URT infection during relapse n (%)	24 (100	26 (92.9)	1.783^a^	0.182
Time (in months) from onset of disease to SSA use	15.07±4.58			

Abbreviation‏: SSA, steroid sparing agents; URT, upper respiratory tract. ^a^Chi-Square test.

**Figure 1:**
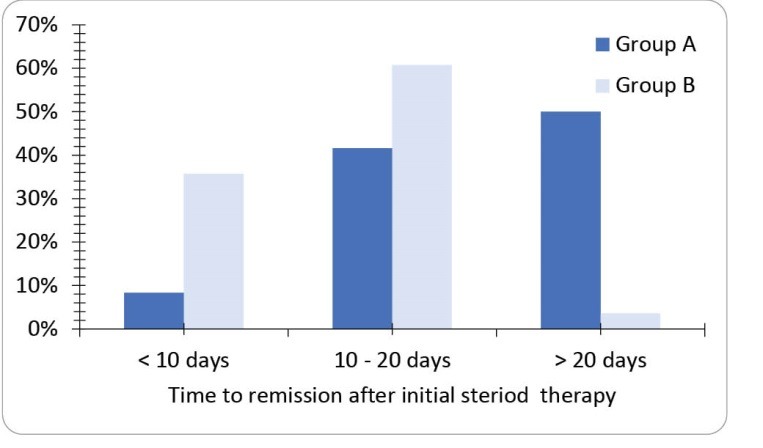
Days to remission after initial steroid therapy.

Figure 1 demonstrates days to remission after initial steroid therapy. In group A, 2 patients (8.3%) get remission in less than 10 days, 10 patients (41.6%) get remission in 10-20 days, and 12 patients (50.0%) needed more than 20 days to get remission. In group B, 10 patients (35.7%) get remission in less than 10 days, 17 patients (60.7%) get remission in 10-20 days and 1 patient (3.6%) needed more than 20 days to get remission. There were significant longer days to remission in group A (*P* = 0.001).

**Figure 2:**
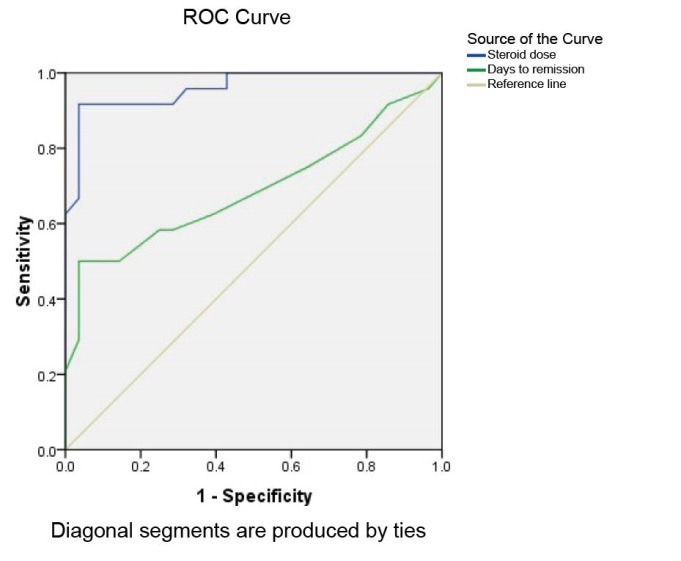
ROC curves of cumulative steroid dose in the first 6 months and days to remission in initial episode nephrotic syndrome. The upper left corner location of the curves indicates a good sensitivity and specificity of test in predicting steroid dependency. The area under the curve (AUC) represents the overall accuracy of the test.

Table 3 and Figure 2 show patients with nephrotic syndrome who required more than 20 days of full dose steroid to achieve remission in initial presentation are prone to steroid dependency with 50% sensitivity and 96.4% specificity. The positive predictive value was 92.3% and negative predictive value was 69.23%. Area under the curve was 0.69

**Table 3:** Prediction of steroid dependency by using 20 days to remission after initial steroid therapy and cumulative steroid dose more than 140 mg/kg received by patients during the first 6 months of treatment

	**Days to remission After initial steroid therapy**	**Cumulative steroid dose**
Sensitivity	50%	91.67
Specificity	96.4%	85.7
PPV	92.3	84.6
NPV	69.23	92.3

Abbreviations: PPV, positive predictive value; NPV, negative predictive value

Cumulative steroid dose > 140 mg/kg/6 months can predict steroid dependency with 91.67% sensitivity and 85.7% specificity. The positive predictive value was 84.6 and negative predictive value of 92.3. Area under the curve was 0.96.

## 5. Discussion

Development of steroid dependency is one of the difficult problems in the management of children with idiopathic nephrotic syndrome. These children are at risk of the complications of long-term steroid treatment and more likely to require cyclosporine as alternative therapy. Identification of such children early in the course of the disease will therefore be useful in counseling parents and alerting the attending clinician to the need to closely monitor these patients.

We found that nephrotic children who required a cumulative steroid dose more than 140 mg/kg to maintain remission during the first 6-month period of treatment are at high risk to require SSA for disease control. Further, in our study patients, those individuals who did not achieve remission by day 20 of the initial prednisone course became steroid dependent with > 96% specificity. Efforts have been made earlier to recognize the relapse pattern of a child presenting with a first manifestation of idiopathic nephrotic syndrome. Constantinescu et al (6) reported in a cohort of 56 North American children that the rapidity of initial response to steroid therapy especially in patients without hematuria are more likely to be infrequent relapses. There analysis was limited to the first year of the disease and most of the patients were treated according to the international study of kidney disease in children (ISKDC) protocol. In our study we could not confirm the relevance of hematuria in predicting idiopathic nephrotic syndrome severity, nor was it confirmed by another study conducted by Yap et al (5). These authors have analyzed their experience in 25 years in a group of 91 Asian children with idiopathic nephrotic syndrome, with a minimum follow up of 6 months. These researchers found that remission after day 9 of the initial prednisone treatment were predictive factors for steroid dependency.

Letavernier et al(7) have reported their experience in 35 patients who were treated with a longer induction schedule of prednisone close to our protocol, with a minimum follow up of 12 months. Steroid dependency developed in their patients who did not achieve remission within 20 days. Our study confirms and expands the results of the above studies. Patients who develop steroid dependency were late responder to steroids in their initial episode and the duration to remission correlate significantly with steroid dependency in all our nephrotic children. However, 12 of 39 patients who went into remission before 20 days, experienced steroid dependency (sensitivity 50%). While 12 of 13 patients who were proteinuric at day 20th, developed steroid dependency (specificity 96.4%). Thus using days to remission after initial steroid administration as a predictive of steroid dependency is a specific but not sensitive test. This means that remission before 20 days does not exclude the possibility of steroid dependency while remission after 20 days confirms the high incidence of steroid dependency.

Factors predicting relapse of nephrotic syndrome are considered risk factors for steroid dependency, as relapse means more steroid to control the disease and this increase the cumulative dose of steroid required. Discontinued steroid usage may decrease the risks for steroid toxicity. However, patients with frequently relapsing nephrotic syndrome may require prednisone doses above the cut-off level for steroid toxicity. It is well-known that disease activity in idiopathic nephrotic syndrome has a natural tendency to decrease over time in most cases (9,10)_._ Disease control may, however, require a more intensive immunosuppression. For steroid-dependent patients, pediatric nephrologists have to make a choice between a steroid-based treatment with the recognized adverse effects and immunosuppressive drugs with a risk of over-immunosuppression and drug-specific side effects, like calcineurin inhibitor-related nephrotoxicity.

Predictive factors for high-degree steroid-dependent forms of idiopathic nephrotic syndrome may consequently be of interest and provide some therapeutic guidelines for the attending physicians. The risk of overestimating steroid dependency appears to be relatively high and it has to be taken into consideration. Such an overestimation of steroid dependency would result to unjustified administration of SSA. In our study, 24 of 52 children with SSNS (46%) required SSA. Our treatment protocol is based on a-4-week prednisone course at disease onset and a maximum daily dosage of 80 mg/m^2^ followed by 1.5 mg/kg alternate day prednisone to be withdrawn to the lowest dose that can keep patients in remission. Lack of drug compliance and the high incidence of acute illnesses predispose to frequent relapsing and steroid dependency. In France and most European countries, they give pulse therapy of methylprednisolone after one month of oral prednisone before considering the patient steroid resistant. Recently, it was detected that the vast majority of patients (83%) who require methyprednisone pulse finally also needed cyclosporine (CyA) to keep remission (7).

The aim of immunosuppressive medications was to taper steroids to a non-toxic levels without disease relapse. The relapse rate has been markedly decreased in children of the present study after receiving SSA. As each relapse is associated with disease-related thrombembolic or infectious risks and an increase of steroid dosage, earlier adequate immunosuppression with stable disease control should lessen disease morbidity. Further, longer periods of high-range proteinuria due to uncontrolled idiopathic nephrotic syndrome may be nephrotoxic and lead to interstitial fibrosis. Except from adequate immunosuppression and disease control, the administration of CyA may have beneficial impacts on disease course. Hoyer et al (11) have detected that an 8-week course of oral CyA at disease onset significantly diminished the relapse rate in the first nine months following disease onset. The risk of unjustified use of CyA for some patients may cause some hesitation. Recent data showed an increased risk for CyA-related renal toxicity if treatment duration exceeds 36 months. These data also indicate that CyA treatment should be avoided if possible in children younger than five years (12). As long as the treatment duration of oral CyA is maintained short (<12 months) and the dosage is ≤5 mg/kg/d, there is currently no evidence of irreversible kidney injury on control biopsies (13)*.*

However, it is well recognized that the most severe cases of steroid-dependent nephrotic syndrome commonly happen in children in the first five years of life (14). Thus, one has to carefully evaluate the benefit risk ratio of CyA treatment in such individuals. Additionally, CyA renal toxicity is at least partially reversible following a short-period of oral CyA treatment, as its main pathophysiological element appears to be prolonged vasoconstriction and not necessarily interstitial fibrosis or arteriolar hyalinosis (15).

The use of mycophenolate mofetil (MMF) instead of CyA in patients who have required methyprednisone pulse should be mentioned. We can report little experience on the use of MMF in steroid-dependent patients. More data are currently available about the effects of MMF in high-degree steroid dependency (16). Hence, an early switch from CyA to MMF and/or a switch from MMF to CyA in the case of relapse could be incorporated in the discussion of future protocols (17).

The mean age of onset of nephrotic syndrome in the present study, was 4.4 years and approximate that of other reports (6). Although earlier studies have shown an increase of steroid dependency with age (18,19). Other studies demonstrated a higher risk of frequent relapse and steroid dependency in nephrotic syndrome children with young age at onset (3,20). The present study did not show any significant association with age at onset of nephrotic syndrome and steroid dependency.

There was no significant difference in steroid dependency between both sexes in our study. However, some studies reported that males were more at risk of developing steroid dependency than females (3,21). This finding may be a consequence of the overall predominance of male patients with nephrotic syndrome, although this seems to be not pronounced in this study. MCD has been thought to be an immune disease connected to seasonal occurrences in atopic individuals (22,23). However, in our study, patient and family history of atopy was found in only about 19% of the patients. In addition, we did not show any significant association between atopy and steroid dependency.

An important finding in the present investigation was the significant correlation of URT infection with relapsing of nephrotic syndrome. It is well known that relapses of MCD may be triggered by episodes of URT infection. All steroid dependent children in this study show relapses associated with URT infections. Although not all non-steroid dependant group show relapse with URT infection, the difference was not significant. However, patients who do not relapse during episodes of URT infection might be less likely to be steroid dependant.

## 6. Conclusions

Cumulative steroid dose in the first six months of treatment and the need of more than 20 days to achieve initial remission can predict steroid dependency in children with nephrotic syndrome.

## Limitations of the study

The study was retrospective and the proportion of patients was relatively few. This may limit generalization of results. Additionally, the retrospective nature of the analysis constitutes a limitation of our study, although these data are difficult to obtain prospectively, because of the small incidence of idiopathic nephrotic syndrome in the pediatric population.

## Authors’ contribution

MAA, ME and HA conducted the design of the research and acquisition of information. NMA, AAE analyzed the data and drafted the manuscript. All authors read, revised, and approved the final manuscript.

## Conflicts of interest

There were no points of conflicts to declare.

## Funding/Support

No fund or support received for this study.
